# Development of a full thickness macular hole after vitrectomy for rhegmatogenous retinal detachment: a sequential study via optical coherence tomography

**DOI:** 10.1186/s12886-018-0932-x

**Published:** 2018-10-11

**Authors:** Hsin-Yu Yang, Chang-Sue Yang

**Affiliations:** 1 Department of Ophthalmology, Taipei Veterans General Hospital, No. 201 Shipai Road, Sec. 2, Taipei, 11217 Taiwan; 2 Department of Ophthalmology, Shin-Kong Wu Ho-Su Memorial Hospital, No. 95, Wen Chang Road, Taipei, Taiwan; 3 Department of Ophthalmology, School of Medicine, Taipei Medical University, Taipei, Taiwan

## Abstract

**Background:**

To demonstrate a full thickness macular hole (MH) development after vitrectomy (VT) for rhegmatogenous retinal detachment (RRD) and to investigate the possible disease mechanism with optical coherence tomography (OCT).

**Case presentation:**

A 47-year-old female underwent 23G vitrectomy surgery to repair the macula-detached RRD successfully. However, intraretinal cysts initially developed two months after surgery. Cysts gradually increased in number and size, and cystoid macular edema was noted at the 5th month. Thereafter, inner retina dehiscence and a lamellar macular hole developed. The lamellar hole further dehisced and progressed into a full-thickness MH at the 10th month. The patient then received 23G vitrectomy and internal limiting membrane peeling surgery. OCT and fundus picture showed macular hole sealed 10 days afterward.

**Conclusions:**

The mechanism of secondary MH included tangential traction, cystoid degeneration of macula, and glial migration. The sequential OCT studies provide evidence to support the disease mechanism of cystoid degeneration of the macula.

## Background

Idiopathic macular hole(MH) was believed to be caused by tangential traction of the posterior hyaloid on the parafoveal area. However, MH development after rhgematogenous retinal detachment(RRD) surgery was rare and the mechanism was not well understood. We reported a case of MH formation after pars plana vitrectomy(VT) for RRD. A complete sequential OCT was performed and supported one proposed mechanism from previous literature. To the best of our knowledge, the following is the first reported case in the literature.

## Case presentation

**Fig. 1:**
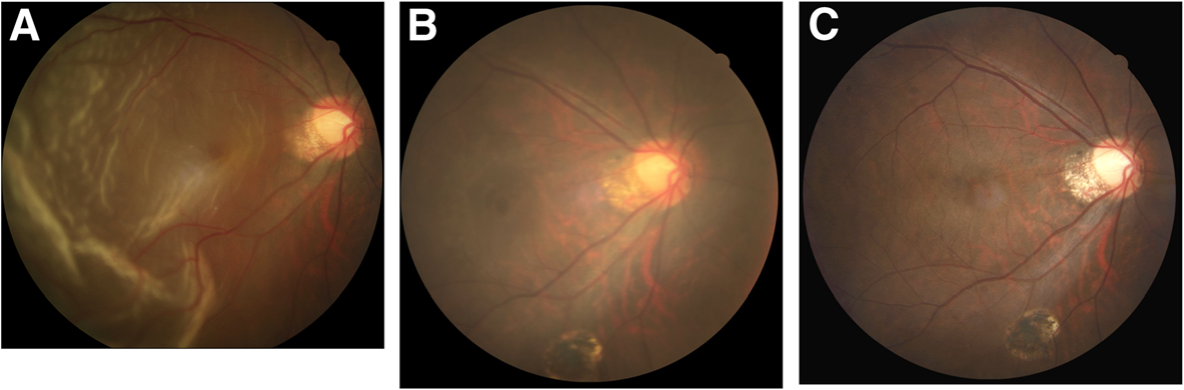
**a** Fundus picture showed macular-off rhegmatogenous retinal detachment from temporal side. **b** Fundus picture revealed macular hole formation with halo and adjacent lamellar hole at the 10th month. **c** Macular hole sealed 10 days after internal limiting membrane peeling surgery

**Fig. 2:**
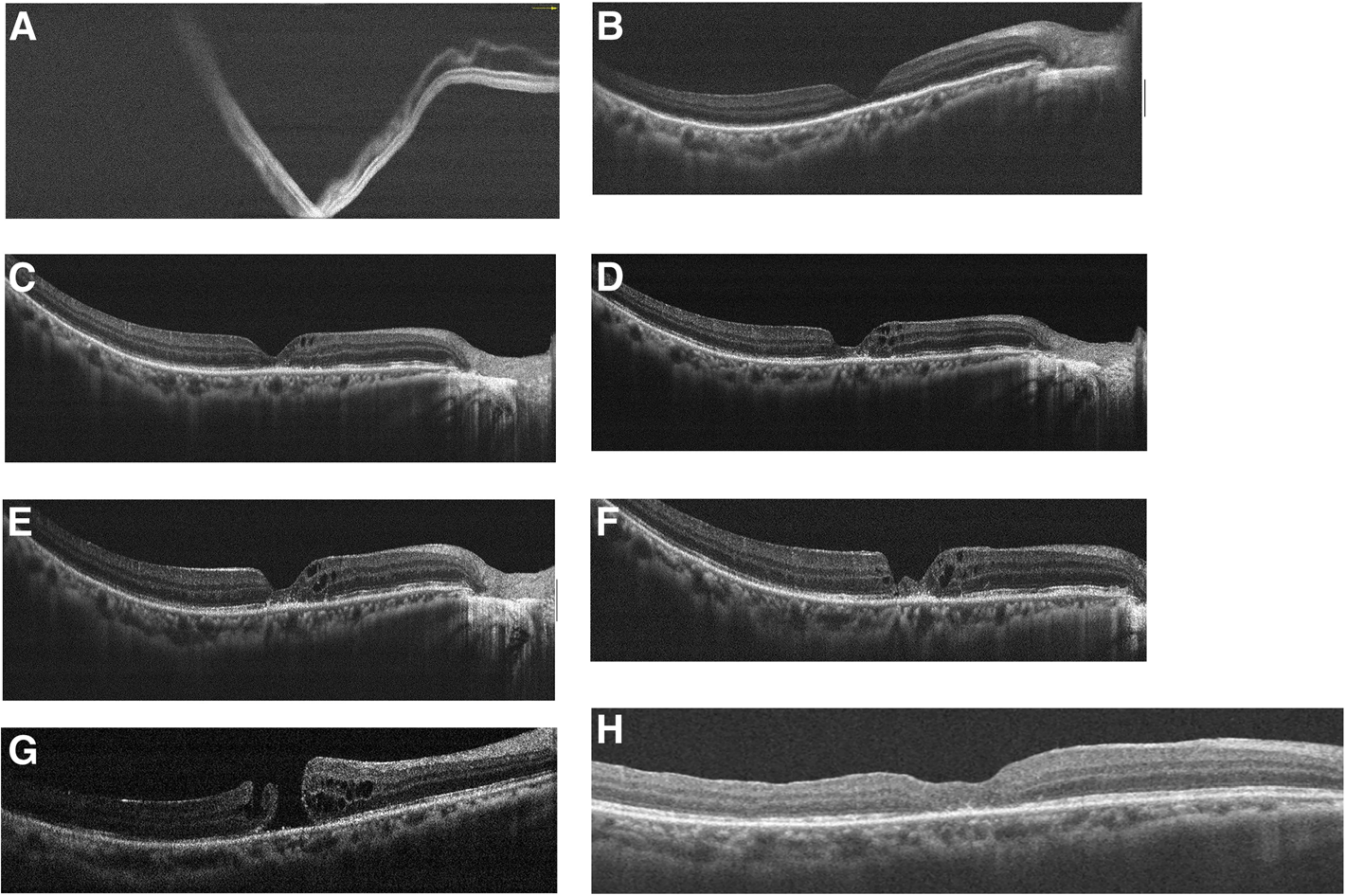
Sequential findings of optical coherence tomography (OCT). VA:visual acuity. (horizontal B-scan passing through the fovea in all OCT images). **a** Macular off rhegmatogenous retinal detachment.(VA was counting fingers in front of 10 cm distance). **b** Attached retina after vitrectomy for repairing rhegmatogenous retinal detachment. Focal ellipsoid zone disruption at macular area was noted. (VA was 4/60). **c** Intraretinal cyst formation was found 2 months after RRD repair surgery. (VA was 4/60). **d** Juxtafoveal intraretinal cysts increased in size and numbers at the 4th month post-operation. (VA was 4/60). **e** At the 5th month after surgery, there was increased intraretinal cystoid change. Topical ketorolac eye drop three times a day was prescribed since then. (VA was 5/60). **f** Lamellar hole developed about half year after RRD surgery. (VA was 5/60). **g** MH formation with halo and adjacent lamellar hole at the 10th month. (VA was 3/60). **h** Successful hole closure after MH repairing surgery. (VA was 6/30)

A 47-year-old female with history of high myopia − 10 diopters presented with acute visual field defect for 2 days. Best-corrected visual acuity of right eye was counting finger in front of 10 cm distance. Both eyes were phakic. Indirect ophthalmoscopy of right eye showed a superotemporal RRD with a tear at 11 o’clock (Fig. 1a). OCT showed compatible result of a bullous macular off RD (Fig. 2a). The 23 gauge pars plana VT, endolaser and gas temponade with 25% SF_6_ were performed. The patient was instructed to maintain prone position for 7 postoperative days. Two weeks after surgery, OCT revealed focal ellipsoid zone disruption at macular area (Fig. 2b). Two months afterward, OCT showed intraretinal cyst formation (Fig. 2c). Visual acuity remained 4/60 over the right eye for four months postoperatively. Progression of juxtafoveal intraretinal cyst was noted after 4 months and increased cystoid change was found at the 5th month (Fig. 2d and e). Topical ketorolac, a kind of Non-Steroidal Anti-Inflammatory Drug was given to the patient three times a day since then. Lamellar hole developed about half year later (Fig. 2f). Finally, OCT and fundus exam demonstrated through MH formation with halo and adjacent lamellar hole at the 10th month (Figs. 1b and 2g). Her visual acuity of right eye remained 5/60 since the fifth month and dropped to 3/60 at the tenth. Thus, patient received 23 gauge pars plana VT, internal limiting membrane peeling, and gas tamponade with 20% SF_6_ 10 months after previous surgery. Successful hole closure was revealed by OCT and fundus exam on the 10th day after second operation (Figs. 1c and 2h). Two years after macular hole surgery, her recent visual acuity recovered to 6/30.

## Discussion

Secondary MH formation following RRD repair surgery was first documented in 1988 [1]. This rare phenomenon carried prevalence varying from 0.32 to 0.96% [2]. Macular-off RRD and repeated surgery for RRD seemed to be important risk factors of this phenomenon [2, 3].

The mechanism of secondary MH included tangential traction, cystoid degeneration of macula, and glial migration.

Tangential traction caused by PVD [2], residual vitreous, and ERM were thought to cause anteroposterior traction of the macula, and lead to MH. However, there were cases of MH formation without evidence of posterior cortical vitreous traction after VT for RRD [4] and even after ERM removal. Thus, tangential traction may not the only factor of secondary MH formation.

Macular cystoid degeneration was proposed as subtle break of internal limiting membrane resulted in hydration of fovea in RRD. Following reattachment of the macula, the edematous retina was then stretched and weakened, leading to MH [3]. Byon et al. assumed previous PVD and macular-off RD causing fragility of foveal tissue, and following phagocytosis of damaged foveal tissue results in MH formation [2]. In the present case, a serial OCT before MH formation was well-documented. Intraretinal cysts initially developed 2 months after RRD repair. Cysts increased in number and size were noted in the following five months. Thereafter, inner retina dehiscence and a lamellar macular hole developed. The lamellar hole further dehisced and progressed into a full-thickness MH at the 10th month. We speculated that macula-off retinal detachment leads to ischemic change and fragility of retinal tissue. Cystoid degeneration with intraretinal edema follows, and then the damaged photoreceptors are phagocytosed by retinal pigmented epithelium. The empty neurosensory space of retina results in macular hole formation. The successive OCT observation in our case supported the pathogenetic mechanism of macular cystoid degeneration theory.

Glial migration may contribute to MH formation as inflammation due to RD can activate glial cell. Afterward, there is glial migration and proliferation followed by contraction of glial plaque surrounding macular area. Then the edges of the umbo dehiscence are pulled outward, finally resulting in MH [4, 5]. Nevertheless, more studies should be provided to prove this speculation.

## Conclusion

We conclude that the sequential OCT provided a non-invasive and useful tool for confirmation of diagnosis, understanding of pathogenesis, and serial anatomical evaluation of MH development after VT for RRD.

## Abbreviations

MH: Macular hole
OCT: Optical coherence tomography
RRD: Rhegmatogenous retinal detachment
VT: Vitrecotmy
